# Dissecting autonomy in a resource-constrained setting: a descriptive qualitative study of women’s decisions on the surgical treatment of early breast cancer in northern Sri Lanka

**DOI:** 10.1080/26410397.2025.2494396

**Published:** 2025-04-16

**Authors:** Ramya Kumar, Gopikha Sivakumar, Dhivya Thuseetharan, Chrishanthi Rajasooriyar

**Affiliations:** a Senior Lecturer, Department of Community and Family Medicine, Faculty of Medicine, University of Jaffna, Jaffna, Sri Lanka.; b Medical Student, Faculty of Medicine, University of Jaffna, Jaffna, Sri Lanka; d Consultant Clinical Oncologist, Teaching Hospital, Jaffna, Sri Lanka; Consultant Clinical Oncologist, Tellippalai Trail Cancer Hospital, Tellippalai, Sri Lanka

**Keywords:** patient autonomy, breast cancer, mastectomy, breast conservation therapy, Sri Lanka

## Abstract

Breast cancer treatment is a contested space in which therapeutic decisions often collide with women’s values and preferences. In northern Sri Lanka, mastectomy remains the mainstay of surgical treatment of early breast cancer (EBC) despite evidence of equivalent survival following breast conserving surgery (BCS) and radiotherapy. This study explores autonomy in decision-making among women with EBC who were eligible for BCS and underwent mastectomy in northern Sri Lanka. A descriptive qualitative study was carried out among 15 women referred for adjuvant therapy to Tellippalai Trail Cancer Hospital in Jaffna district after having a mastectomy for EBC. Participants were recruited between January and May 2022 until data saturation was reached. Data were gathered through semi-structured interviews, which were transcribed in Tamil, translated into English, coded using QDA Miner Lite software, and analysed thematically. Women’s autonomy in EBC treatment decisions is limited by various factors in northern Sri Lanka. The hospital setting is not conducive to informed decision-making, and women do not receive sufficient information. Neither survival rates nor risks/benefits of the surgical options are discussed in a systematic way. Although many women appear to be satisfied with their involvement in decision-making, their decisions are guided by incomplete information and fears of spread/recurrence communicated by treating teams. In the absence of policies and protocols to support patient autonomy, women “choose” the more invasive option: mastectomy. While it behoves medical professionals to provide evidence-based information, governments and the global health community must support strengthening healthcare systems to advance women’s health and rights in lower-resource settings.

## Plain language summary

In northern Sri Lanka, mastectomy is the most common surgery for early breast cancer. But evidence shows the same survival outcomes after less invasive breast conserving surgery and radiotherapy. In this article, we explore autonomy in decision-making among women with early breast cancer in northern Sri Lanka. These women would have been eligible for breast conserving surgery. We interviewed 15 women who had a mastectomy for early breast cancer and were referred to a cancer centre in Jaffna district for further management. The interviews were held in Tamil, transcribed and then translated into English. The data were analysed using qualitative software and organised into themes. The findings suggest that women in northern Sri Lanka have limited space to make their own decisions on treatment of their early breast cancer. The hospital setting is not conducive to women making informed decisions. Most women do not receive enough information about the treatment options available. Although many women seem to be satisfied with their involvement in decision-making, their decisions are guided by incomplete and biased information provided by treating teams. Medical professionals must provide accurate and comprehensive information to patients. Governments and the global health community should support strengthening healthcare systems to promote women’s health and rights in lower-resource settings.

## Résumé

Le traitement du cancer du sein est un espace disputé dans lequel les décisions thérapeutiques se heurtent souvent aux valeurs et préférences des femmes. Dans le nord du Sri Lanka, la mastectomie reste le pilier du traitement chirurgical du cancer du sein au stade précoce, malgré des données prouvant une survie équivalente après une chirurgie conservatrice du sein et une radiothérapie. Cette étude explore l’autonomie de décision des femmes atteintes d’un cancer du sein au stade précoce qui étaient éligibles à une chirurgie conservatrice et ont subi une mastectomie au Sri Lanka septentrional. Une étude qualitative descriptive a été réalisée auprès de 15 femmes adressées pour un traitement adjuvant au Tellippalai Trail Cancer Hospital, dans le district de Jaffna, après avoir subi une mastectomie pour un cancer du sein au stade précoce. Les participantes ont été recrutées entre janvier et mai 2022 jusqu’à saturation des données. Les données ont été recueillies au cours d’entretiens semi-structurés qui ont été transcrits en tamoul, puis traduits en anglais, codés à l’aide du logiciel QDA Miner Lite et analysés thématiquement. L’autonomie des femmes dans les décisions relatives au traitement du cancer du sein au stade précoce est limitée par plusieurs facteurs dans le nord du Sri Lanka. Le milieu hospitalier n’est pas propice aux décisions éclairées et les femmes ne reçoivent pas suffisamment d’informations. Ni les taux de survie ni les risques/avantages des options ne sont abordés de manière systématique. Bien que beaucoup de femmes semblent satisfaites de leur participation à la prise de décision, leurs décisions sont guidées par des informations incomplètes et par les craintes de propagation/récidive communiquées par les équipes soignantes. En l’absence de politiques et de protocoles pour soutenir l’autonomie des patientes, les femmes « choisissent » l’option la plus invasive: la mastectomie. Alors qu’il incombe aux professionnels médicaux de fournir des informations fondées sur des données probantes, les autorités et la communauté mondiale de santé doivent soutenir le renforcement des systèmes de santé pour faire progresser la santé et les droits des femmes dans les environnements à faibles ressources.

## Resumen

El tratamiento del cáncer de mama es un espacio disputado donde las decisiones terapéuticas a menudo chocan con los valores y las preferencias de las mujeres. En el norte de Sri Lanka, la mastectomía continúa siendo la base del tratamiento quirúrgico del cáncer de mama en etapa temprana (CMET) a pesar de la evidencia de supervivencia equivalente después de una cirugía para conservar el seno (CCS) y radioterapia. Este estudio explora la autonomía en la toma de decisiones entre mujeres con CMET que eran elegibles para CCS y se sometieron a una mastectomía en el norte de Sri Lanka. Se llevó a cabo un estudio cualitativo descriptivo con 15 mujeres referidas para terapia adyuvante a *Tellippalai Trail Cancer Hospital* en el distrito de Jaffna después de someterse a una mastectomía por CMET. Las participantes fueron reclutadas entre enero y mayo de 2022 hasta que se alcanzó la saturación de datos. Los datos fueron recolectados por medio de entrevistas semiestructuradas, que fueron transcritas en tamil, traducidas al inglés, codificadas utilizando software *QDA Miner Lite* y analizadas temáticamente. La autonomía de las mujeres en sus decisiones sobre el tratamiento de CMET está limitada por diversos factores en el norte de Sri Lanka. El ámbito hospitalario no es propicio para una toma de decisiones informada y las mujeres no reciben suficiente información. Ni las tasas de supervivencia ni los riesgos/beneficios de las opciones son discutidos de manera sistemática. Aunque muchas mujeres parecen estar satisfechas con su participación en la toma de decisiones, sus decisiones son guiadas por información incompleta y por temores de extensión/recurrencia comunicada por los equipos de tratamiento. A falta de políticas y protocolos que apoyen la autonomía de las pacientes, las mujeres “eligen” la opción más invasiva: mastectomía. Si bien es la obligación de profesionales médicos proporcionar información basada en evidencia, los gobiernos y la comunidad de salud mundial deben apoyar el fortalecimiento de los sistemas de salud para promover la salud y los derechos de las mujeres en entornos con escasos recursos.

## Introduction

The management of breast cancer has evolved in the last two decades, with advances in cancer diagnosis and therapeutics giving way to an array of treatment options. The primary treatment for early breast cancer is surgery followed by a single or a combination of adjuvant therapies including radiotherapy, chemotherapy, endocrine therapy, and targeted therapy.^1^ While treatment regimens are tailored based on tumour-node-metastasis staging, hormone receptor status and expression of other targetable receptors, mastectomy and breast conserving surgery (BCS) are the two main surgical options for the primary tumour.^2^

Breast-conserving therapy (BCT), which includes BCS and adjuvant radiotherapy, remains the standard of care for early breast cancer,^2^ with evidence of equivalent survival and local recurrence rates and better cosmetic outcomes compared to mastectomy.^3–6^ Even so, mastectomy rates have not declined globally, with recent spikes in bilateral mastectomy reported from many settings.^7–9^ While introducing BCS in lower-resource settings has been challenging owing to the lack of trained personnel, technologies, and equipment,^10^ mastectomy rates are high in Asia where facilities exist.^8,11^ In Sri Lanka, where this study is set, only 32% of women with early breast cancer were treated with BCT between 2016 and 2020; 62% underwent modified radical mastectomy and the remainder (6%) did not undergo surgery.^12^

Making decisions on breast cancer treatment is not easy, given the complexity of a cancer diagnosis, the range of treatment regimens available, and the rapidly evolving landscape of breast cancer care.^13^ Studies conducted primarily in higher-resource settings suggest that most women with early breast cancer receive information to support decision-making^14^ although the information conveyed is of variable quality and often partial, with many women not comprehending the risks, benefits, and outcomes of BCS when they undergo mastectomy.^15,16^ It is reported that women’s decisions are heavily swayed by the surgical team,^14^ and that in some instances, women delegate decision-making authority to their surgeons.^13^

Autonomy, understood as a patient’s right to freely make informed decisions based on their values and preferences, is a core ethical principle in medical practice.^17^ However, the assumption that human beings are capable of making rational and independent decisions when provided adequate information has been contested, giving way to a relational approach that considers the sociocultural context in which patients make decisions and the myriad of factors that shape decision-making.^18,19^ The latter include socio-economic disparities, access to health information and services, health facility policies and guidelines, the doctor–patient relationship, the space for patients to challenge treatment decisions, and gendered norms and assumptions about health and the body.^20^

A relational approach recognises that autonomy-supportive conditions can be fostered within healthcare systems. Here, the concept of shared decision-making, which refers to clinicians and patients making decisions collaboratively based on the best available evidence, has gained traction.^21^ Shared decision-making is associated with better adherence to treatment, less unwarranted intervention, and greater patient satisfaction with cancer care.^21,22^ Certain surgical units that practice shared decision-making report higher BCS rates than those that do not.^14^ Supporting patient autonomy in breast cancer treatment decisions involves not only ensuring the provision of accurate information about the treatments, their risks/benefits, and outcomes, but also building the infrastructure and resources needed to create conducive decision-making environments.^13,20,23,24^

The limited research on autonomy in breast cancer treatment decisions in lower-resource settings focuses almost entirely on the extent to which women participate in decision-making. For instance, research from India and Malaysia suggests that surgeons and family members play a crucial role in decision-making.^25–27^ Little is known about the decision-making context and the resources and systems available to support women to exercise their autonomy in breast cancer care. Addressing this gap, this study explores autonomy in decision-making among women with early breast cancer, paying attention to the conditions under which women who were eligible for BCS decided to undergo mastectomy at a cancer care centre located in Jaffna district in post-war northern Sri Lanka.

## Breast cancer care in northern Sri Lanka

Sri Lanka has experienced a long-standing economic crisis that worsened after the country defaulted on its external debt in 2022. The public healthcare system has been crippled by insufficient financial and other resources, yet continues to deliver services on a non-fee levying basis, albeit subject to availability. A fee-for-service private sector operates in parallel and remains the only alternative when public sector services are in short supply or not available.^28,29^

Within the public system, breast cancer care is delivered through specialist medical and surgical oncology units established across the country. Many vacancies exist in these units owing to the outmigration of health professionals, which has intensified since 2022. Radiation facilities are in short supply in the system as a whole, albeit sufficiently available in Jaffna district. Screening mammography is not performed routinely due to the unavailability of machines in most districts. While clinic-based breast examination is accessible at community clinics, women are also encouraged to perform breast self-examination.^30^ Advanced diagnostic services (e.g. molecular diagnostics) and newer modalities of cancer treatment (e.g. immunotherapy, some targeted therapies) are currently not available at public hospitals. While these services are available in the private sector on a fee-levying basis, access is limited to those who can afford private healthcare.

Sri Lanka’s Northern Province (population 1.2 million) came out of a protracted civil war in 2009. Most women diagnosed with breast cancer in the province seek treatment at the Tellippalai Trail Cancer Hospital (TTCH) in Jaffna District – one of five districts that make up the Northern Province. As the centre of excellence in cancer care in the north, TTCH began to offer precise adjuvant radiotherapy in 2019, when the facility acquired a linear accelerator. Digital mammography, which is needed for diagnosis and post-treatment surveillance, became available at Teaching Hospital Jaffna (THJ), the nearest tertiary care centre, that same year. While THJ has two surgical oncology units with two surgical oncologists (one in each), breast cancer surgery is also offered through the general surgical units at THJ and other hospitals in the district. The entire Northern Province is serviced by four clinical oncologists, of whom three practice in both TTCH and THJ.

Referral pathways for breast cancer in the north are similar to other parts of the country. When a breast lump/lesion is identified, women generally present (or are referred by a general practitioner) to the outpatient department of a public hospital or to a surgeon in the private sector. They usually receive surgical treatment at a general surgical or surgical oncology unit at a public hospital, with a minority accessing the private sector for surgical care. In the event that a breast lesion is found to be malignant, women are referred for oncological care. Surgical treatment decisions are made primarily by surgeons, at times in consultation with an oncologist. Due to resource constraints, multidisciplinary meetings are not routinely held, except for complex cases, as is the practice elsewhere in Sri Lanka.

## Methods

This exploratory descriptive qualitative study was conducted at the outpatient department of TTCH.

Women ≥18 years who were eligible for BCS, and underwent mastectomy for early-stage breast cancer within six months of the data collection period and presented to the outpatient department of TTCH for adjuvant therapy, were recruited to the study.

Eligibility for BCT was evaluated by the attending consultant oncologist (CR) following thorough review of patient records. A total of 35 patients were enrolled for curative intent treatment during the recruitment period (January-May 2022). Among them, mastectomy was indicated for 10 owing to skin involvement, multicentric disease or multifocal microcalcification in other quadrants. Of the remaining 25 patients, five (20%) had BCS, one insisted on mastectomy at diagnosis, and the surgical team recommended mastectomy for one patient because she was unlikely to comply with radiotherapy and regular follow-up. The remaining 18 patients were invited to the study by nurses who were not involved in the research project. They were requested to return to the clinic for an interview on a specified date and informed that they would be compensated for travel.

Data were generated with semi-structured in-depth interviews, carried out by two female medical students (DT and GS) in the fourth year of training. They were members of the research team and had no direct contact with the participants before data collection. They were trained in qualitative interview techniques by two experienced qualitative researchers (CR and RK). Practice interviews were conducted with health personnel acting as simulated patients. All interviews took place on weekends, in the outpatient department of TTCH when clinics were not in session. Both data collectors participated in all interviews, taking turns interviewing and note-taking.

The interviews were held in Tamil, the local language, and facilitated with an interview guide developed by the research team. The guide was designed to explore what participants knew about the surgical treatment options for breast cancer before they underwent mastectomy, the information given by healthcare providers, and how they selected mastectomy as their treatment option. Informed written consent was obtained from all participants by DT and GS at the time of interview. The interviews lasted between 22 and 56 minutes and were digitally recorded with prior consent. Data saturation was reached after 15 interviews.

The recorded interviews were transcribed into Tamil by a native Tamil speaker. DT and GS, who are bilingual native Tamil speakers proficient in English, translated the transcripts into English and anonymised them by removing all content that could potentially identify participants. Translations were meticulously reviewed and compared with the audio recordings by CR who is a native Tamil speaker. The translated transcripts were then scrutinised by RK and CR and preliminary themes identified. A structured coding tree was developed, containing pre-defined codes based on the study objectives complemented with *in vivo* codes drawn from the preliminary themes. The transcripts were then coded using QDA Miner Lite software by DT and GS and reviewed by CR and RK. A deductive thematic analysis was performed by RK and CR guided by three broad questions: under what circumstances women made decisions; to what extent evidence-based information was provided; and in what ways were women supported to make decisions based on their values and preferences. At least two research team members reached consensus at each stage of the analysis. The results were not shared with the participants for feedback.

The research team had to contend with their positionalities from the outset. RK is an academic with training in medicine and public health, CR is a consultant clinical oncologist at TTCH and THJ, and GS and DT are medical students. CR decided to conduct the study after she was approached by surgeons trained in BCS at THJ who were keen to see a change in practice. It was previously believed (based on anecdotal evidence) that eligible patients refused BCS fearing radiotherapy, but a formal study had not been undertaken. CR conceptualised the study to identify the reasons for refusal of BCS and invited RK onboard. As passionate advocates for women’s rights, we reflected on the power differentials between the medical establishment, which we were part of, and our prospective participants. We believed two medical students in their fourth year of training, who had completed their clinical rotations in oncology, would be sufficiently familiar with the research topic yet not perceived to be members of the treating team by participants. Therefore, we invited GS and DT to collaborate with us on the project.

On completion of the study, we published a first paper on information gaps on BCS and their consequences for women with early breast cancer. It became clear during the analysis phase that the problems were not limited to a fear of radiotherapy but were far more complex and layered. We felt a second publication devoted to concerns of autonomy and patient rights was needed, given the implications of the findings. As CR was involved in the care of some of these patients, RK, who is not involved in the delivery of cancer care, led the analysis and writing of this paper.

Ethics approval was obtained (28 December 2021) from the Ethics Review Committee of the Faculty of Medicine, University of Jaffna, Sri Lanka (Ref. No. J/ERC/21/128/NDR/0260).

## Results

Fifteen women who were eligible for BCS and underwent mastectomy for early-stage breast cancer participated in the study. Their demographic details are depicted in Table 1.

**Table 1.** Demographic details of participants (*n* = 15).

	No.	%
Age (years)		
<50	04	27
50–59	06	40
60 and over	05	33
Marital status		
Married	13	86
Widowed	01	07
Separated/divorced	01	07
Educational level		
Primary or below	05	33
Lower secondary	04	27
Upper secondary	05	33
Diploma^a^	01	07
Total	15	100

^a^ There were no degree holders in the sample.

The findings are presented under three themes that correspond to the three questions that guided the analysis: (1) unsupportive decision-making environments; (2) partial information on treatment options; and (3) women’s preference or “Hobson’s choice”?

The lengthier direct Tamil to English translations of terms used by participants to describe breast cancer treatment are replaced in the quotations with “BCS,” “mastectomy,” “radiotherapy,” “chemotherapy” for the sake of brevity.

### Unsupportive decision-making environments

Most women spoke of making treatment decisions in consultation with a surgeon or the surgical team. However, consultation times were limited and one-to-one consultations usually not possible, except in the private sector. Pre-surgical counselling at public hospitals was brief, usually lasting 10–15 minutes, and mostly held during a busy clinic or ward round. As P5 explained: “*They would have spent about 10 minutes with me at the clinic, two to three days before the surgery*.” For P4, time was even shorter: “*The chief surgeon and the senior registrar spoke to me for about five minutes, after which I agreed for mastectomy*.”

In some instances, women were hastily informed of the decision made on their behalf by the treating team. P14 believed she was scheduled for BCS until the night before her surgery when she was shocked to hear otherwise: “*They told me that the cancer had spread … and that they would have to remove the whole breast. But I was told on the night before the surgery … the assisting doctor spoke to me during the ward rounds … just for about five minutes*.” P3’s family was informed of the cancer diagnosis during the initial consultation, but she was left in the dark: “*I got to know I had breast cancer only after I was admitted to the ward for surgery. The doctor spent about 15 minutes talking to me during the ward round*.”

Most notably, none of the patients spoke of having had structured meetings with their surgeons/surgical teams or any other efforts to convey information in a comprehensive way. Most women had consented for mastectomy during a brief consultation with their surgeon, with many denying having received information from other ward staff, including junior doctors and/or nurses. A few had received bits of information from other healthcare providers, in an ad hoc manner. For instance, P11’s surgeon informed her about her treatment plan, and she received additional information from a junior doctor during the informed consent procedure. Some women, like P3, did not feel comfortable asking questions: “*I did not clear my doubts or ask questions because I was scared*.” On the other hand, P4 felt it was not feasible for doctors to have lengthy discussions with patients: “*They explained whatever they could within the short period available* * … * *I am not disappointed because they explained things patiently without getting irritated, even during the ward rounds*.”

Despite the rushed and seemingly unfavourable circumstances under which women participated in making decisions on their surgical treatment, few identified the lack of information and time constraints as problems. Indeed, many were satisfied with the information they received. As P7 noted, “*The information provided was adequate. The decision was up to us*.” However, as we will see next, the information they received was insufficient and often misleading.

### Partial information on treatment options

Most women did not receive accurate information regarding the surgical treatment options available for the treatment of early breast cancer. Of 15 participants, nine were either not aware of BCS or did not know they were eligible for BCS before their mastectomy.

Some participants had not been informed that their breast could be conserved. As P5 explained, “*The doctors told me I had a lump in the breast and that they would remove the whole breast … they did not say anything about removing just the lump.*” P10 shared a similar experience: “*They did a biopsy first and told me that the lump appeared to be cancer and that I had to undergo surgery. They said the surgery would be big and that I should have the whole breast removed*.” P11, while not given specific information about the two options, recalled that the option of removing only the lump had been mentioned by her surgeon in the course of pre-surgical counselling: “* … I was told that if I get only the lump removed, then I might need to undergo further surgery, if the tumour spreads*.”

Many were informed that they were at an advanced stage of breast cancer that precluded BCS as a surgical option. The surgeon let P1 know that she was too late for BCS: “*If I had come earlier, they would’ve been able to do BCS* * … * *but when we showed up, it was very late, and the lump had already become large*.” The surgical team had discussed BCS as a potential treatment option for P12 but changed their mind after the MRI scan: “*[The surgeon] said the cancer had spread deep inside and they would do a mastectomy because there was a risk of [the cancer] spreading further*.” Surgical teams also recommended mastectomy due to axillary involvement, for instance, in P14: “*They told me that the cancer had spread to the lymph nodes in my arm and that they would have to remove the whole breast*.” P13, whose surgeon had recommended mastectomy for similar reasons, feared the consequences of BCS: “*I didn’t urge the doctor to perform BCS because I felt I might have to suffer later*.”

Open discussions regarding the risks and benefits of the two surgical procedures were generally not held, except to communicate unfounded warnings about BCS. As P1 explained, “*I was not informed about the outcomes, possible complications, benefits and disadvantages of BCS and mastectomy*.” For others like P2, the risks and benefits of BCS were conveyed in partial ways: “*I was told that the longer-term outlook would be better with mastectomy and that I would live longer. If I go for BCS, if the cancer happens to spread again, it can spread very quickly … .*” Survival rates following the two procedures were generally not discussed. “*They did not tell me anything about how long I would live after the surgery* * … *,” P5 said. P11 pointed to similar information gaps: “*They didn’t comment on survival, the consequences of not removing the breast, the possible complications of the surgery or the follow up plan* * … .*” Overall, P6 knew very little about her treatment plan at the time of surgery: “*They did not give any information, except that I had cancer in my breast and that the whole breast needed to be removed*.” Although P1 was aware of BCS when she had a mastectomy, she was not aware that survival rates were equivalent for the two options: “*I recently met a few patients who had undergone BCS and were on radiotherapy … I didn’t realize [BCS] could also be done on me, and that the outcome would be more favourable*.”

Information about the complications of mastectomy were conveyed, in some instances. P4 and P10 knew about the possibility of limb swelling, before their mastectomy. The surgeon informed P10 that she may not be able to use her arm after surgery and asked her to give consent if she “wished to take the risk.” P6 was advised that she may not have sensation on the chest wall after mastectomy. Some were counselled on post-surgical rehabilitation. Exercise was recommended for P11 by the surgical team: “*They advised me to do regular limb exercises without being confined to a seat*.” P7 was encouraged to work but within limits: “*They asked me to be cautious about lifting heavy weights and such*.” Noteworthy is that cosmesis was discussed only with one participant, P2: “*After the surgery, [the doctor] informed me about artificial breasts and advised me to come for follow up treatment*.”

Most women were not aware before their mastectomy that they may need adjuvant therapy. This information was usually conveyed after surgery, perhaps because the finer details of these treatments are decided based on tumour pathology, receptor status, and advanced imaging (bone scan and CT scan) done post surgically. Right after her mastectomy, P2 was informed that she would be referred to an oncologist: “*But the surgeon didn’t mention anything regarding radiotherapy or chemotherapy*.” The nuts and bolts of cancer treatment were usually explained by the clinical oncologist on presentation for adjuvant therapy at TTCH. “*After seeing the [histology] report, the oncologist reassured me that the remnants of the lump were not visible and that they would give chemotherapy in case there were invisible cancer cells in the rest of the body*,” P4 elaborated. P5 became aware of the details when she met the oncologist at the clinic at THJ: “*They told me at the Jaffna Hospital (THJ) that I will need eight sessions of chemotherapy … * *they may take further measures depending on my condition. I asked if I should undergo radiotherapy, and they said no*.”

A few participants believed providing too much information to patients could be detrimental. “*The surgeon informed me about the overall picture of the treatment, that was sufficient. If I had been given details about the complicated treatment, I would have been afraid*,” P2 said. P12 felt the surgeon could have spent more time with her, but was uncertain about the extent of information she would have liked to receive: “*If they had informed us regarding chemotherapy, we would’ve been afraid. So, it’s better to avoid giving such information beforehand*.” However, it would seem that the information provided was partial and led women to select mastectomy, as discussed next.

### Women’s preference or “Hobson’s choice”?

The majority of participants felt they had been sufficiently involved in the decision-making process and that their preferences were taken into consideration. However, on closer analysis, it seems that many women would actually have preferred BCS had they not received biased information from their treating teams. In the end, the explicit and implicit warnings against BCS left women with no choice but to select mastectomy – a “Hobson’s choice” of sorts.

*“I was given the freedom to choose between BCS and mastectomy by the surgeon, and I asked him if I could go for BCS, as I thought it would be difficult for me to go out as usual, if I had a mastectomy. But he said that since it had already spread to the axillary region, and since my child is still very young, it would be better if I chose to remove the whole breast …” * (P2)

P15 had hoped that only her lump would be removed until she met her surgeon. At the hospital, her surgeon asked her whether she preferred mastectomy or BCS, albeit with a caveat: “*I was told that the cancer won’t spread to other sites if I had mastectomy … .*” P2’s surgeon recommended mastectomy citing the possibility of the cancer spreading if “*the tumour was not removed completely*.” P4 claimed she was given a “choice” but was informed that the cancer may recur or spread to other sites and require additional surgical intervention, if she had BCS. P10 shared a similar story: “*[The surgeon] told me that the entire breast should be removed because if the lump was removed alone, the cancer might spread to other areas*.” When P8 asked her surgeon if only the lump could be removed, the surgeon warned her that BCS would involve radiotherapy. For P7, it was an oncologist who recommended mastectomy:

* “… [the oncologist] told me that since I was young it would be easier to have the entire breast removed, to prevent the spread of the cancer. [They] told me that removing the whole breast was better, but I was free to choose whichever option I preferred. They did not force me.”* (P7)

The absence of any real “choice” was patently obvious when we enquired as to whether their decision would be different if given a second chance. Most participants indicated they would select mastectomy again based on the “fact” that it was the better option for malignant breast disease. Indeed, it was a no-brainer for many. As P3 succinctly stated, “*If there is a possibility of spread, obviously I would opt for removal of the whole breast*” – as had been conveyed by her surgeon. P7 reaffirmed she would undergo mastectomy if it meant that the “whole cancer” would be destroyed – as was conveyed to her. For P10, the decision was entirely hers and she would make it again based on the risk of spread with BCS, as was communicated by her surgeon. P11 clearly indicated that she would prefer to remove only the lump but went with mastectomy because it was the “better option.” Having been informed that her cancer had spread, P12 indicated her preference for mastectomy, which she believed was better in such instances.

A minority of women were indignant that their preferences went unheeded by their treating teams. For P8, the decision had been made unilaterally by her surgeon despite her repeated requests for BCS:

“*I repeatedly asked if I could get the lump removed instead of the whole breast, but the doctor insisted that the latter was the better option. If I had BCS, I would have to undergo continuous radiotherapy and there is a risk of spread, he said. He was decisive about the treatment option (mastectomy) for me. … I was not happy with the final decision. This decision was made only because the doctor said so.*” (P8)

Others also regretted the decision. As P4 explained, “*If I’d been informed properly with sufficient information, I would’ve opted for BCS*.” P5 said she would select BCS if given a second chance: “*Having one breast without the other is useless, they might as well have removed both breasts … the doctors should have given me this option.*” P14 felt betrayed because she had believed she would have BCS until the night before the surgery:

“*I thought they would remove just the lump and that was what I wanted. … . Then they told me  … they would have to remove the whole breast. ..I did not know what to do … . [The surgical team] mostly discussed among themselves … They could have at least spoken to my family.*” (P14)

## Discussion

The space for women to exercise their autonomy in treatment decisions for early breast cancer seems limited in northern Sri Lanka. Most study participants made decisions under unsupportive conditions with little or no access to evidence-based information. While the decision-making environment did not encourage asking questions or challenging decisions made by treating teams, many participants accepted the lack of information and non-involvement in decision-making as the norm. Despite the importance of these contextual factors, the literature on autonomy in breast cancer care primarily focuses on the extent to which women are involved in decision-making, specifically who makes decisions and why (see Liu et al.^14^). Underpinned by individualist notions of self-determination, this traditional approach to autonomy has been critiqued for downplaying social and other conditions that structure decision-making in healthcare settings.^18^ A relational interpretation, by contrast, demands that we consider the extent to which patients are supported to make decisions, based on their values and preferences, by trained health professionals, guided by policies, protocols, and supported with the resources needed to advance patient autonomy.^13,20,31^

Supportive clinical communication involves listening to patients, making them feel their concerns are heard, encouraging them to ask questions, and empowering them with the knowledge they need to make informed decisions.^32^ Our findings suggest that none of these areas of clinical communication were addressed in intentional and systematic ways when information on breast cancer treatment options was conveyed in the clinical setting in northern Sri Lanka. Treatment teams frequently pushed women to select mastectomy, often placing them in a “Hobson’s choice” situation. The *Guidelines on Ethical Conduct for Medical and Dental Practitioners Registered with the Sri Lanka Medical Council*^33^ require doctors to “provide sufficient details and information in non-technical language the [*sic*] nature, purpose and material risk of the proposed procedure to enable the patient to form a proper decision” (p. 68). However, such codes of conduct that depend on self-regulation may be inadequate to hold healthcare providers accountable to their patients. In the United States, patient autonomy is supported by legislation that mandates medical professionals to provide complete and accurate information,^34,35^ while in the United Kingdom, patient advocacy groups help dissatisfied patients to navigate the complex terrain of complaint procedures within the National Health Service (see Voiceability^36^, as an example). In Sri Lanka and most other lower-resource settings, such avenues remain underexplored.

Laws, policies and protocols to support patient autonomy must be enacted/formulated in tandem with efforts to improve health systems infrastructure and resources. If governments are to respect, protect and fulfil the right to health, it is imperative that they strengthen the capacity of healthcare systems to support women to be more involved in breast cancer treatment decisions.^37^ The right to health, as interpreted by the United Nations’ Committee on Economic Social and Cultural Rights (CESCR), addresses four key areas, namely the availability, accessibility, acceptability, and quality (3AQ) of healthcare services.^38^ A rights-based approach to breast cancer care would demand governments to ensure that the required services are available and accessible. Here, accessibility encompasses physical, economic, and *information* accessibility. The services delivered must also be acceptable to the community, *respecting its needs, values, and the broader sociocultural context*, and of sufficient technical quality.^38^ In other words, adopting a rights-based approach to health would necessitate supporting patient autonomy and shared decision-making in cancer care.

In northern Sri Lanka, at the time of writing, 16 surgeons and four oncologists provide breast cancer surgery and adjuvant therapy, respectively, to a population of approximately 1.2 million. The preliminary results of a study carried out among 15 surgeons performing breast cancer surgery in northern Sri Lanka indicate that limited infrastructure and training gaps contribute to the high rate of mastectomy in the province.^39^ Ideally, in the tertiary care setting, treatment decisions should be supported by multidisciplinary teams (MDT) involving a range of specialists. According to the European Society of Breast Cancer Specialists (EUSOMA), a breast centre (i.e. an institution where breast cancer is diagnosed and treated) should have a team of core members, including a breast radiologist, breast radiographer, breast pathologist, breast surgeon, breast medical oncologist, breast radiation oncologist, breast care nurse, and a breast data manager. Extended members of such MDTs, to whom patients could be referred if the need arises, include psycho-oncologists, physiotherapists, and prosthetic specialists, among others.^40^ This range of expertise is not available in Sri Lanka, where surgeons and oncologists take on all this work in addition to a heavy caseload that is not limited to breast cancer.^41^ Currently, Sri Lanka has only 25% of the required number of oncologists per population based on global standards, compelling clinicians to manage four times the recommended number of patients. Consequently, only the more challenging cases are handled by MDTs. In addition, the limited infrastructure at public hospitals poses a significant challenge to clinicians who would like to have one-to-one consultations with patients. Most clinics are held in large open rooms where multiple doctors see different patients simultaneously, with attendant concerns of privacy and confidentiality.

Given the resource constraints at tertiary care centres, particularly after the economic crisis, integrating cancer care into the primary care system may be an avenue that could be explored. At present, cancer care remains outside the primary care system, aside from a few cancer prevention interventions like cervical cancer screening and clinical breast examination. As reflected in the findings, pre-surgical counselling for early breast cancer took place only in busy hospital settings or during (brief) private sector consultations. Ideally, a woman with breast pathology should be able to discuss their diagnosis and treatment plan with a trusted physician trained in supportive clinical communication. A primary care physician would be ideally placed to do so although, at present, most medical officers and “general practitioners” practising in the public and private sectors in Sri Lanka, are not trained in general practice. As well, general practitioners need to be empanelled, and health information and referral systems strengthened, to support shared care between general practitioners and oncology teams.

Studies on healthcare decision-making in lower-resource settings suggest that gender norms, patriarchal family structures, community beliefs, and taboos around illness and end of life, influence women’s healthcare decisions.^42^ We too have highlighted the role families play in breast cancer treatment decisions in a prior publication based on the same study.^43^ Indeed, the bioethics literature questions the relevance of individualistic notions of patient autonomy in more “collectivist” cultures where autonomy may be deferred to spouses or other family members.^44^ While acknowledging the importance of such cultural differences, other scholars critique the individualist-collectivist binary that serves to normalise withholding information from patients and deferring decisions to family members.^45^ They suggest that medical hierarchies and class- and gender-based power differentials between doctors and their patients may support this paternalistic approach towards patient care. In other words, assumptions about so-called collectivist cultures may reinforce oppressive power relations that disempower patients, while detracting from the urgent need to institute mechanisms to hold healthcare providers accountable to their patients. In our study, the few women who expressed indignation about not having received information about BCS before their mastectomy, evidently had no recourse to redress.

In settings where breast cancer care is prioritised, women’s health activism has played a crucial role in drawing attention and resources to breast cancer.^46,47^ In the United States, the 1970s saw a shift in treatment from radical mastectomy to less invasive surgical procedures, at a time when breast cancer survivors were calling out the lack of information and support for women having radical mastectomies.^16,47^ Feminists in the 1980s exposed the sexualised nature of breast cancer treatment and questioned the emphasis placed on prosthesis and breast reconstruction over women’s wellbeing.^48,49^ These advocacy efforts came to fruition in a well-funded breast cancer research programme launched in the 1990s.^46,47^ An example from a lower-resource setting, the Uganda Women’s Cancer Support Organization (UWOCASO), established in 2004 by a group of breast cancer survivors, supports women with breast cancer and engages in advocacy efforts in Uganda. Over 200 cancer survivors currently serve as UWOCASO volunteers, providing information and supportive care for survivors and their families.^50^ Through persistent advocacy, the organisation has obtained seats in key health policymaking bodies in Uganda.^51^

While there are lessons to be learnt from these organising efforts, advocacy in low- and middle-income countries (LMICs) is constrained by the limited resources available to develop cancer care beyond awareness and screening programmes.^51^ Research on breast cancer in these settings has a narrow focus on risk factors, screening behaviours, treatment adherence, and behavioural change.^52,53^ This emphasis is entrenched by a global health paradigm that normalises vastly different healthcare standards and inequities in access between and within countries.^54,55^ While governments of LMICs have a crucial role to play in allocating resources to build healthcare systems, the inequitable distribution of health resources, including migration of health professionals, must also be addressed at the global level.

This study comes with some limitations. First, data were gathered in a hospital setting by medical students, which may have placed restrictions on patients sharing their views freely. However, the outpatient department at TTCH is quiet on weekends when clinics are not in session and the data collectors were fairly junior and had no role in the delivery of cancer care. Second, we explored patient autonomy from the perspective of women, neglecting the perspective of surgeons. Soon-to-be published work, already communicated in an abstract by Naganathan et al.^39^ involving CR, explores breast surgical oncology from the perspective of surgeons in the Northern Province and will complement the results of this study. A third limitation is that we did not recruit the fifth of patients who selected BCS during the study period. A follow-up study with them may help to shed light on how women could be better supported to select the less invasive treatment option, BCS.

## Conclusion

Patient autonomy in surgical treatment decisions for early breast cancer appears to be limited in northern Sri Lanka. While the hospital setting is not conducive to making informed decisions, women do not receive sufficient information to guide their decisions. The provision of complete and accurate information to women with breast cancer, needs to be institutionalised and supported by patient autonomy-strengthening policies and shared decision-making protocols within healthcare facilities. Such measures should be combined with efforts to retain health professionals, build infrastructure and introduce MDTs in the local setting. The existing system of general practice could be revamped to integrate supportive cancer care with primary care. Doing so would relieve tertiary care centres of at least part of their workload, increasing the time available to surgeons, oncologists, and others, to support women to be more involved in their breast cancer care. To this end, the government must allocate more resources to strengthen the healthcare system, and the global health community should call for strengthening cancer care to advance women’s health and rights in lower-resource settings.

## Provenance statement

This article was not commissioned and went through external peer review.
